# Pretreatment Resistance to Integrase Inhibitors in the Swiss HIV Cohort Study From 1995 to 2025

**DOI:** 10.1093/ofid/ofag435

**Published:** 2026-07-17

**Authors:** Tom Loosli, Marco Labarile, Nanina Anderegg, Paul Frischknecht, Nuri Han, Sandra E Chaudron, Gilles Wandeler, Marcel Stöckle, Matthias Cavassini, Alexandra Calmy, Patrick Schmid, Luigia Elzi, Irene A Abela, Michael Huber, Karoline Leuzinger, Sabine Yerly, Matthieu Perreau, Huldrych F Günthard, Roger D Kouyos

**Affiliations:** Department of Infectious Diseases and Hospital Epidemiology, University Hospital Zurich, Zurich, Switzerland; Institute of Medical Virology, University of Zurich, Zurich, Switzerland; Department of Infectious Diseases and Hospital Epidemiology, University Hospital Zurich, Zurich, Switzerland; Institute of Medical Virology, University of Zurich, Zurich, Switzerland; Department of Infectious Diseases and Hospital Epidemiology, University Hospital Zurich, Zurich, Switzerland; Department of Infectious Diseases and Hospital Epidemiology, University Hospital Zurich, Zurich, Switzerland; Department of Infectious Diseases and Hospital Epidemiology, University Hospital Zurich, Zurich, Switzerland; Institute of Medical Virology, University of Zurich, Zurich, Switzerland; Department of Infectious Diseases and Hospital Epidemiology, University Hospital Zurich, Zurich, Switzerland; Institute of Medical Virology, University of Zurich, Zurich, Switzerland; Department of Infectious Diseases, Inselspital, Bern University Hospital, University of Bern, Bern, Switzerland; Division of Infectious Diseases and Hospital Epidemiology, University Hospital Basel, University of Basel, Basel, Switzerland; Division of Infectious Diseases, University Hospital Lausanne, University of Lausanne, Lausanne, Switzerland; Division of Infectious Diseases, University Hospital Geneva, University of Geneva, Geneva, Switzerland; HOCH Health Ostschweiz, St Gallen, Division of Infectious Diseases, Infection Prevention and Travel Medicine, Department General Internal Medicine, St Gallen, Switzerland; Division of Infectious Diseases, Ente Ospedaliero Cantonale, Ospedale Regionale di Bellinzona e Valli, Bellinzona, Switzerland; Faculty of Medicine, University of Basel, Basel, Switzerland; Department of Infectious Diseases and Hospital Epidemiology, University Hospital Zurich, Zurich, Switzerland; Institute of Medical Virology, University of Zurich, Zurich, Switzerland; Institute of Medical Virology, University of Zurich, Zurich, Switzerland; Clinical Virology, University Hospital Basel, Basel, Switzerland; Laboratory of Virology, Geneva University Hospitals, Geneva, Switzerland; Division of Immunology and Allergy, Lausanne University Hospital and University of Lausanne, Lausanne, Switzerland; Department of Infectious Diseases and Hospital Epidemiology, University Hospital Zurich, Zurich, Switzerland; Institute of Medical Virology, University of Zurich, Zurich, Switzerland; Department of Infectious Diseases and Hospital Epidemiology, University Hospital Zurich, Zurich, Switzerland; Institute of Medical Virology, University of Zurich, Zurich, Switzerland

**Keywords:** HIV, INSTI resistance, transmitted resistance

## Abstract

**Background:**

Studies in Switzerland and other settings have reported minimal pretreatment integrase strand transfer inhibitor (INSTI) resistance, but many were conducted before widespread use of INSTIs or are limited regarding data availability and representativeness. Consequently, many questions remain regarding the transmissibility of INSTI drug resistance mutations (DRMs).

**Methods:**

In sequences predating antiretroviral therapy (ART) initiation collected in the Swiss HIV Cohort Study (SHCS) resistance database, we assessed INSTI DRMs using the Stanford HIV database algorithm. We use bayesian regression models to assess INSTI DRM trends, as well as epidemiological clustering. Models are adjusted for age, sex, HIV subtype, and time as a spline, with random effects for the SHCS center of registration and the sequencing laboratory.

**Results:**

In total, 3704 SHCS participants had an integrase sequence before ART initiation; 213 (5.8%) had INSTI DRMs, including 11 (0.3%) with major INSTI DRMs. The most common DRMs were the polymorphic accessory INSTI DRMs E157Q, T97A, and L74M. There was no change in the probability of detecting INSTI DRM(s) over the past 3 decades. Epidemiological clustering was similar between individuals with or without pre-ART INSTI DRMs; however, the clusters they formed were smaller, particularly before 2010.

**Conclusions:**

This study demonstrates that in Switzerland pre-ART INSTI DRMs are very rare, despite INSTIs being used for more than 10 years. In cases where pre-ART INSTI DRMs are detected, information on the context in which they emerged is rarely available, highlighting the need for continued genetic surveillance, particularly in the face of increasing numbers of individuals with INSTI resistance globally.

## Teaser

We observed constant low prevalence of integrase strand transfer inhibitor (INSTI) resistance mutations in treatment-naive people with HIV in the Swiss HIV Cohort Study over 3 decades, despite massively increasing INSTI use, suggesting no ongoing INSTI resistance transmission in Switzerland.

Second-generation integrase strand transfer inhibitors (INSTIs) have become part of the commonly preferred antiretroviral therapy (ART) regimen in Switzerland in the last decade due to their high efficacy, favorable adverse effect profile, and high genetic barrier to drug resistance. INSTI resistance is rare in settings such as Switzerland; in a large cohort collaboration, only 7.2% of individuals with viremia on dolutegravir-based ART had major INSTI drug resistance mutations (DRMs) [1]. However, recent studies from low- and middle-income countries report increasing numbers of individuals with high-level resistance to second-generation INSTIs [2, 3]. It is currently unclear how such DRMs affect the transmissibility of HIV-1 strains [3, 4].

Pre-ART INSTI resistance in Switzerland had previously been assessed by Scherrer and colleagues [5] up to 2014. They found that the prevalence of INSTI DRMs was very low, as was the population-level viral load among individuals on INSTI-based ART in the Swiss HIV Cohort Study (SHCS), suggesting a low potential source of transmissible INSTI DRMs in Switzerland. Since 2014, the number of individuals on INSTI-based ART has increased substantially (Supplementary Figure 1), as has the proportion of individuals with non-B HIV subtypes [6], thus representing a change in both the Swiss HIV epidemic as well as the ART landscape.

More recent studies assessing pre-ART INSTI resistance, performed in the MeditRes consortium in France, Greece, Italy, Portugal, and Spain, found 8 of 2705 ART-naive individuals with major INSTI DRMs in 2018–2021; in Poland, Mielczak and colleagues [7, 8] reported 4 of 882 ART-naive individuals between 2016 and 2023 with major INSTI DRMs. In the United Kingdom, Mbisa and colleagues [9] found that among 640 individuals with recent HIV infections between 2014 and 2016, none had major INSTI DRMs at variant frequencies >20%; when considering low-frequency mutations (2%–20%), however, they could identify major INSTI DRMs in 25 individuals. In a systematic review published in 2021, Bailey and colleagues [10] describe the prevalence of transmitted INSTI resistance, and they detected no association with calendar time, HIV subtype, or geographic region but an unexplained increase of polymorphic INSTI DRMs over time.

Studies assessing pre-ART INSTI resistance usually include heterogeneous data collection sources and/or uncertain country representativeness, or limited sample size [7–10]. With a globally increasing number of individuals who have INSTI DRMs while being viremic, systematic surveillance is increasingly relevant [2]. Here, we use pre-ART integrase sequences of individuals participating in the SHCS [11], which is representative of people with HIV on ART in Switzerland, to assess trends in pretreatment INSTI resistance in Switzerland over 3 decades.

## METHODS

### SHCS and Linked HIV Drug Resistance Database

The SHCS is a national observational cohort with semiannual study visits enrolling people living with HIV in Switzerland since 1988, and it covers over 70% of all individuals on ART in Switzerland [11]. The linked HIV drug resistance database collects sequences from SHCS participants from genotypic resistance testing at diagnosis or for treatment management, as well as sequences generated in the context of scientific studies, and it is linked to the SHCS next-generation sequencing database [12]. The SHCS has been approved by the local ethics committees responsible for the participating centers, and all enrolled individuals gave informed consent for study participation, including the use of biological and clinical data for SHCS projects [11].

### Study Population

The study population includes all SHCS participants with an HIV sequence containing integrase from RNA, sampled before or up to 30 days after initiating ART. Data used in this study included all data available by 24 September 2025. For participants with multiple sequences meeting these criteria, we considered the earliest time point.

### Drug Resistance Interpretation and Definitions

Mutations resulting in amino acid substitutions conferring drug resistance will be referred to as DRMs, according to the Stanford HIV database [13]. Identification and categorization of INSTI DRMs, as well as identification of polymorphic INSTI DRMs, was done according to the Stanford HIV drug resistance algorithm, version 9.8 [13]. Because the analysis was restricted to consensus HIV sequences generated from plasma RNA, apolipoprotein B mRNA editing catalytic polypeptide-like (APOBEC)-mediated artifactual detection of INSTI DRMs from proviral DNA was considered unlikely [14, 15].

### Statistical Analysis

We separately estimated the probability of detecting any major or accessory INSTI DRM, any nonpolymorphic INSTI DRM, any polymorphic INSTI DRM, and any major INSTI DRM using bayesian logistic regression implemented in the brms package (version 2.22.0) [16]. Calendar time was modeled as a penalized spline, with age and sex included as covariates. To account for unobserved heterogeneity in the data, we included random effects for the sequencing laboratory and the SHCS center of first registration.

To investigate the impact of HIV subtype and race/ethnicity, we fitted a complementary model in which calendar-time splines were allowed to vary by HIV subtype and race/ethnicity through an interaction term, while including main effects of subtype, race/ethnicity, sex, and age and the same random-effects structure mentioned above. In a supplementary analysis, we assessed the interaction between HIV subtype and race/ethnicity with time but included calendar-time as a linear term.

To explore temporal patterns of specific INSTI DRMs, separate bayesian logistic regression models were fitted for each mutation occurring >10 times, using mutation presence or absence as the binary outcome; covariates and random effects were the same as listed above.

To investigate whether individuals with pre-ART INSTI DRMs differ in terms of epidemiological background, we made use of protease and reverse transcriptase (PR-RT)–sequence-based epidemiological clustering of SHCS participants, described in detail by Labarile and colleagues [17]. We thus modeled the probability of connecting to an existing cluster at the time of HIV diagnosis, using the same bayesian models as described above. We allowed the calendar time spline to vary by the presence of INSTI DRMs. Furthermore, we modeled network connectivity (ie, the number of connections to other SHCS participants), using a zero-inflated negative binomial specification with the same covariate structure. In a supplementary analysis, we investigated all epidemiological clusters to which SHCS participants with pre-ART major INSTI DRMs are connected.

For all statistical models, we report population-average temporal trends. We obtained posterior expected probabilities from the fitted models at the population level—that is, excluding random effects. We computed predictions at 100 equally spaced time points between the earliest and most recent sample in the dataset for all individuals, using their observed covariate values (age and sex), and we averaged predictions across individuals for each posterior draw. For models including HIV subtype and race/ethnicity, we standardized predictions by computing predictions for all individuals at all levels of these covariates. We calculated outcome probability quantiles from the resulting posterior distributions.

All analyses were performed with R software, version 4.3.3. Bayesian models were estimated using the cmdstanr backend (version 0.8.0) with adapt_delta settings between 0.98 and 0.99 to ensure stable sampling. We used weakly informative priors for all model parameters; a description of all models and full prior specifications are provided in the Supplementary Materials. Prior-predictive checks confirmed that these priors produced plausible outcome probabilities (Supplementary Figure 2).

## RESULTS

### Study Population

**Figure 1.**
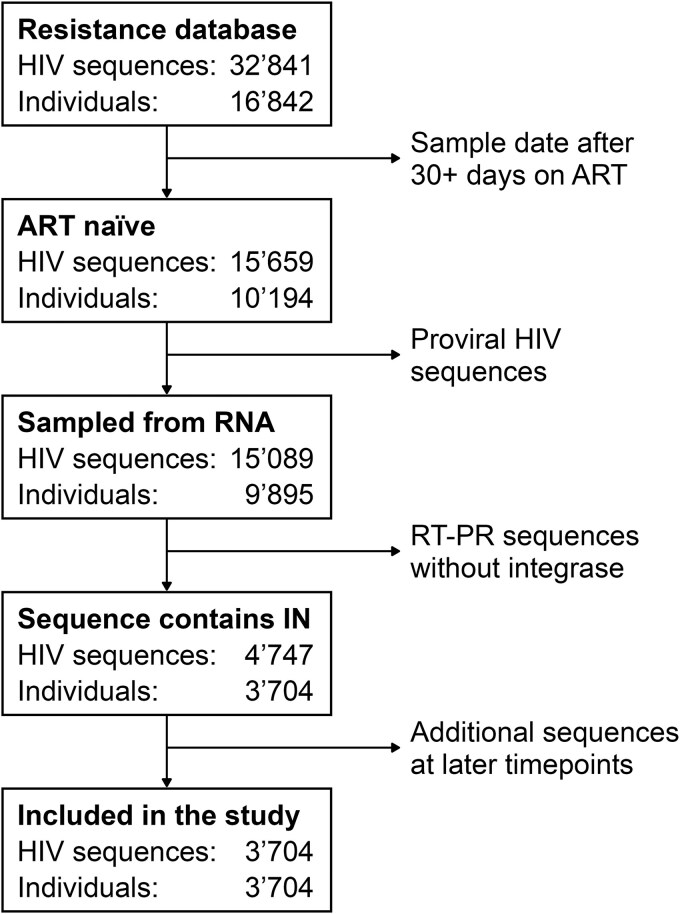
Flowchart describing sequence selection. From the Swiss HIV Cohort Study (SHCS) resistance database, sequences were selected from RNA sampled before antiretroviral therapy (ART) or after up to 30 days on ART including integrase. If multiple sequences for a single individual were available, the sequence from the earliest sampling date was considered. Abbreviations: HIV, human immunodeficiency virus; PR-RT, protease and reverse transcriptase.

By 24 September 2025, the SHCS resistance database included 32 841 HIV sequences from 16 542 SHCS participants. Sequences from samples predating ART initiation or taken within the first month of ART are available for 10 194 individuals. This population is broadly comparable to the total SHCS population but slightly underrepresents individuals with HIV transmission via injecting drug use who registered in the very early years of the cohort (Supplementary Table 1); most (9895 participants [97.1%]) (Supplementary Figure 3*A*) had sequences generated from HIV RNA. Among those, integrase is covered in 3704 individuals (Figure 1). Integrase availability among pre-ART sequences became predominant in 2003. The earliest sequence was sampled on 20 July 1995, and the most recent sequence on 3 September 2025; the median sampling year was 2011 (Supplementary Figure 3*B* and 3*C*).

The 3704 SHCS participants included in this study were mostly male and mostly White (79.9%), with a median age of 37 years at the time of sampling, reflecting the overall SHCS population. The most common HIV subtype was B (57.9%) (Table 1).

**Table 1.** Population Characteristics^a^

Characteristic	Participants, No. (%)^b^		
	No INSTI DRMs (n = 3491)	≥1 INSTI DRM (n = 213)	Overall Study Population (N = 3704)
Sex			
Male	2871 (82.2)	152 (71.4)	3023 (81.6)
Female	620 (17.8)	61 (28.6)	681 (18.4)
Age at time of sample, median (IQR), y	37.0 (30.0–46.0)	36.0 (31.0–46.0)	37.0 (30.0–46.0)
Race/ethnicity			
White	2824 (80.9)	134 (62.9)	2958 (79.9)
Black	332 (9.5)	53 (24.9)	385 (10.4)
Asian	142 (4.1)	11 (5.2)	153 (4.1)
Hispano-American	180 (5.2)	11 (5.2)	191 (5.2)
Other	10 (0.3)	3 (1.4)	13 (0.4)
Missing	1 (0.0)	1 (0.5)	2 (0.1)
Unknown	2 (0.1)	0 (0)	2 (0.1)
Transmission group			
Homosexual contacts	2049 (58.7)	99 (46.5)	2148 (58.0)
Intravenous drug use	185 (5.3)	9 (4.2)	194 (5.2)
Heterosexual contacts	1090 (31.2)	90 (42.3)	1180 (31.9)
Other	167 (4.8)	15 (7.0)	182 (4.9)
SHCS center of registration			
Zurich	1684 (48.2)	91 (42.7)	1775 (47.9)
Basel	441 (12.6)	32 (15.0)	473 (12.8)
Bern	342 (9.8)	13 (6.1)	355 (9.6)
Geneva	211 (6.0)	23 (10.8)	234 (6.3)
St Gallen	161 (4.6)	6 (2.8)	167 (4.5)
Lausanne	587 (16.8)	42 (19.7)	629 (17.0)
Lugano	65 (1.9)	6 (2.8)	71 (1.9)
Sequence sample date, median (IQR)	2011 (2007–2016)	2012 (2008–2015)	2011 (2007–2016)
First VL, median (IQR), log_10_ copies/mL^c^	4.8 (4.2–5.4)	4.7 (4.0–5.3)	4.8 (4.2–5.4)
Missing	456 (13.1)	27 (12.7)	483 (13.0)
First CD4 cell count, median (IQR), cells/µL^c^	378.0 (218.0–552.5)	363.0 (205.5–536.5)	378.0 (218.0–552.0)
Missing	484 (13.9)	26 (12.2)	510 (13.8)
Year of ART initiation , median (IQR)	2012 (2008–2016)	2013 (2009–2015)	2012 (2008–2016)
Time to ART initiation, median (IQR), mo	0.7 (0.1–8.1)	0.8 (0.2–6.2)	0.7 (0.1–7.9)
HIV subtype			
B	2056 (58.9)	87 (40.8)	2143 (57.9)
01_AE	239 (6.8)	4 (1.9)	243 (6.6)
A	173 (5.0)	10 (4.7)	183 (4.9)
C	153 (4.4)	14 (6.6)	167 (4.5)
02_AG	98 (2.8)	25 (11.7)	123 (3.3)
G	56 (1.6)	19 (8.9)	75 (2.0)
Recombinant	583 (16.7)	40 (18.8)	623 (16.8)
Other^d^	79 (2.3)	10 (4.7)	89 (2.4)
Unknown	54 (1.5)	4 (1.9)	58 (1.6)

Abbreviations: ART, antiretroviral therapy; DRMs, drug resistance mutations; HIV, human immunodeficiency virus; INSTI, integrase strand transfer inhibitor; IQR, interquartile range; SHCS, Swiss HIV Cohort Study.

^a^Baseline characteristics of study participants with a pre-ART sequence from HIV RNA including integrase.

^b^Data represent no. (%) of participants unless otherwise specified.

^c^Within 365 days of sampling.

^d^Other subtypes include F (n = 58), D (n = 27), H (n = 2), HIV-1 O group (n = 1), and J (n = 1).

### Detected INSTI DRMs

**Figure 2.**
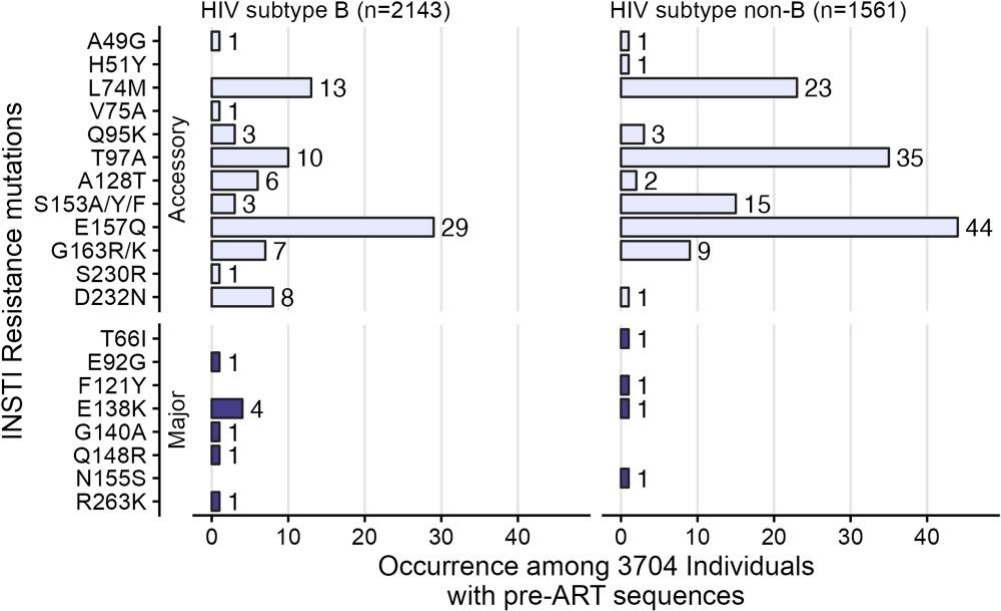
Integrase strand transfer inhibitor (INSTI) drug resistance mutations (DRMs) detected in pre–antiretroviral therapy (ART) sequences. Colors correspond to category major and accessory, according to the Stanford HIV database classification. Note that DRMs occurring in mixed forms were simplified to the DRM. L74M, T97A, and E157Q are considered polymorphic mutations. Abbreviation: HIV, human immunodeficiency virus.

Pre-ART INSTI DRMs were detected in 213 of 3704 participants (5.8%). Most had accessory INSTI DRMs (203 of 213 [95.3%]). Combinations of INSTI DRMs were rare; 198 of 213 participants (93.0%) had a single INSTI DRM. INSTI DRMs at exclusively nonpolymorphic positions were found in 71 participants (33.3%). We identified only 11 participants with major INSTI DRMs. Among them, we observed a wide range of different DRMs. Only E138K, a major INSTI DRM with limited impact on INSTI susceptibility [18], was observed in multiple individuals (n = 5; 4 with HIV subtype B and 1 with HIV subtype D) (Figure 2). Overall, the most frequently observed mutations were E157Q (n = 73 [34.3%]), T97A (n = 45 [21.1%]), and L74M (n = 36 [16.9%]) (Figure 2); all 3 are considered polymorphic INSTI DRMs [19]. E157K, the most commonly observed pre-ART INSTI DRM, was increasing until 2015, after which the estimated observation probability stabilizes at about 2%. Among the other INSTI DRMs occurring at least 10 times, we did not detect a change in the observation probability over time, but credibility intervals were relatively wide due to the limited sample size (Supplementary Figure 4).

Among the 11 participants with major INSTI DRM(s), 1 also had the major protease inhibitor (PI) DRM D30N, which is reported to affect only nelfinavir [20]. Three had additional nucleoside/nucleotide reverse-transcriptase inhibitor DRMs, which included the thymidine analogue mutation K219R, and thymidine analogue mutation revertant T215D in 2 individuals, 1 of which also had S68G, a natural polymorphism that may improve replicative fitness of viruses with K65R [21, 22]. Nonnucleoside reverse-transcriptase inhibitor (NNRTI) DRMs were also detected in 4 individuals and included E138EA (a polymorphic mutation with minimal impact on NNRTI efficacy) [23], P236L (reported to not have any impact on current NNRTIs) [24], E138K (a nonpolymorphic mutation selected under rilpivirine and etravirine) [25, 26], and E138G (an accessory nonpolymorphic mutation selected by etravirine) combined with Y181F (a rare nonpolymorphic mutation that typically represents a revertant) [23, 27]. For 3 of those 11 participants, additional sequences sampled before ART initiation were available; the major INSTI DRM observed in the first sequence persisted only in a participant with E138K. In the other 2 participants, with R263RK or F121FY in the first sequence, the major INSTI DRM was not observed anymore in the second pre-ART sequence (Supplementary Table 2).

### INSTI Resistance Trends

**Figure 3.**
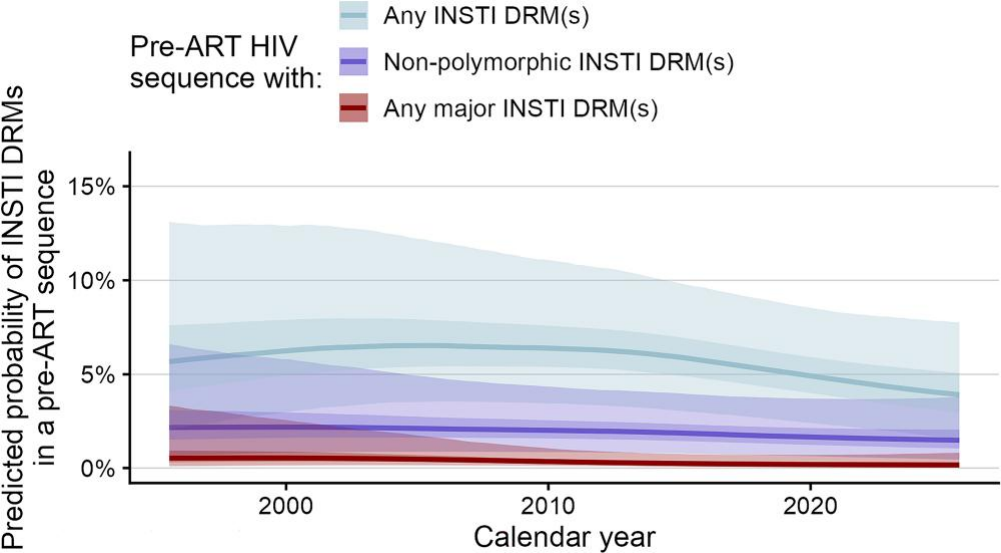
Population trend in integrase strand transfer inhibitor (INSTI) drug resistance mutations (DRMs) over time. Three bayesian regression models were fitted separately for an individual having any INSTI DRMs, INSTI DRMs on strictly nonpolymorphic positions, or any major INSTI DRMs. Calendar time was modeled with a penalized spline, and the model was adjusted for age and sex, including random effects for the sequencing laboratory and the Swiss HIV Cohort Study center of first registration. Solid line represents the posterior median of the expected probability; darker band, the 50% credible interval; and lighter band, the 95% credible interval. Group-level effects were marginalized; uncertainty reflects the mean expectation rather than individual-level variability. Abbreviations: ART, antiretroviral therapy; HIV, human immunodeficiency virus.

The probability of detecting INSTI DRMs in pre-ART HIV sequences remained stable over the past 3 decades in the SHCS, despite the strongly increasing number of individuals on INSTI-based ART since 2014 (Supplementary Figure 1). When considering only INSTI DRMs at nonpolymorphic positions, or only major INSTI DRMs, we find lower but similarly constant probabilities over time (Figure 3). We also do not observe changes in the probability of pre-ART polymorphic INSTI DRMs (Supplementary Figure 5).

**Figure 4.**
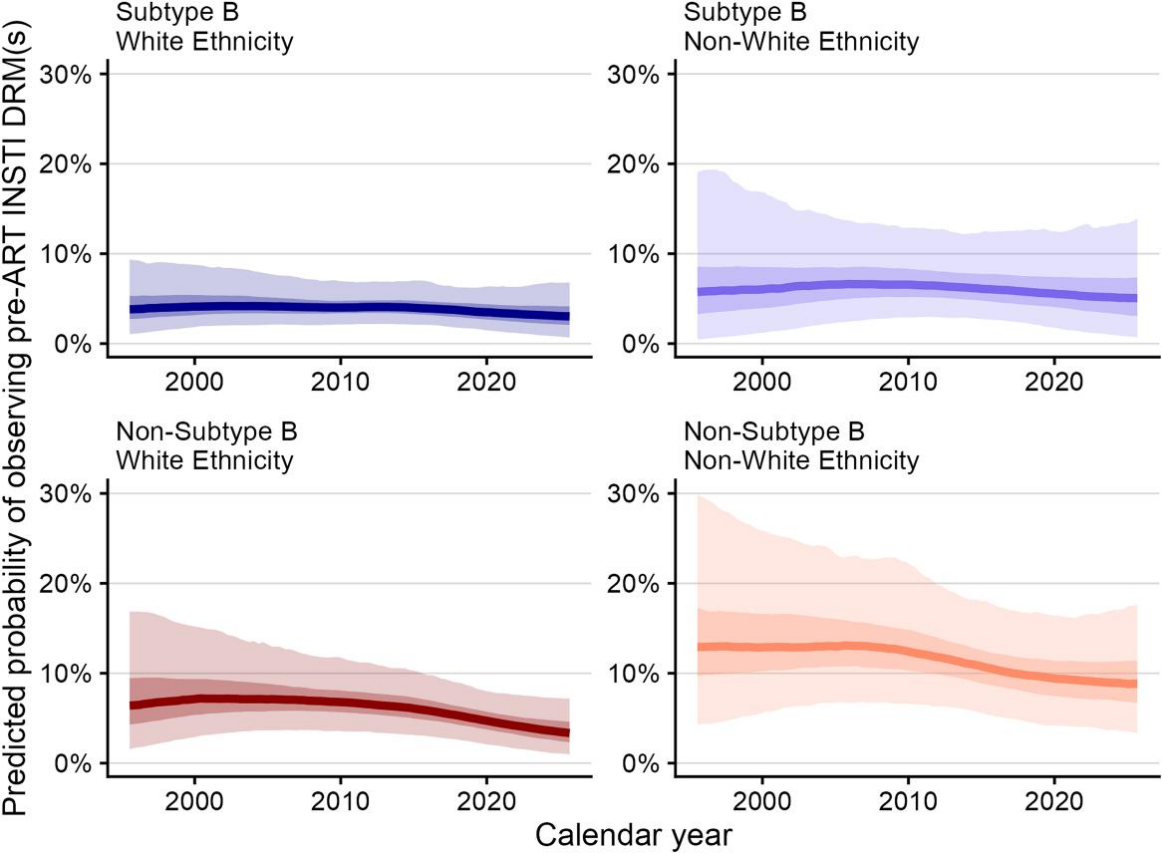
Population trend in any integrase strand transfer inhibitor (INSTI) drug resistance mutations (DRMs) over time by human immunodeficiency virus (HIV) subtype and race/ethnicity. A bayesian regression model was fitted for having any major or accessory INSTI DRMs. Calendar time was modeled with a penalized spline allowed to vary by HIV subtype and race/ethnicity through an interaction term, while including main effects of HIV subtype and race/ethnicity. The model was further adjusted for age and sex, including random effects for the sequencing laboratory and the Swiss HIV Cohort Study center of first registration. Solid line represents the posterior median of the expected probability; darker band, the 50% credible interval; and lighter band, the 95% credible interval. Group-level effects were marginalized; uncertainty reflects the mean expectation rather than individual-level variability.

Although the probability of observing INSTI DRMs is higher in non-White SHCS participants with non-B HIV subtypes, there is no evidence for a change over time (Figure 4). Even when assessing calendar time as a linear variable, we do not detect differences in the slopes by HIV subtype and race/ethnicity (Supplementary Table 3). With restriction to nonpolymorphic INSTI DRM positions, we find lower overall probabilities and similarly stable trends; with fewer cases, differences between groups become less pronounced, and uncertainty increases (Supplementary Figure 6).

### Trends in Sequence-Based Clustering

**Figure 5.**
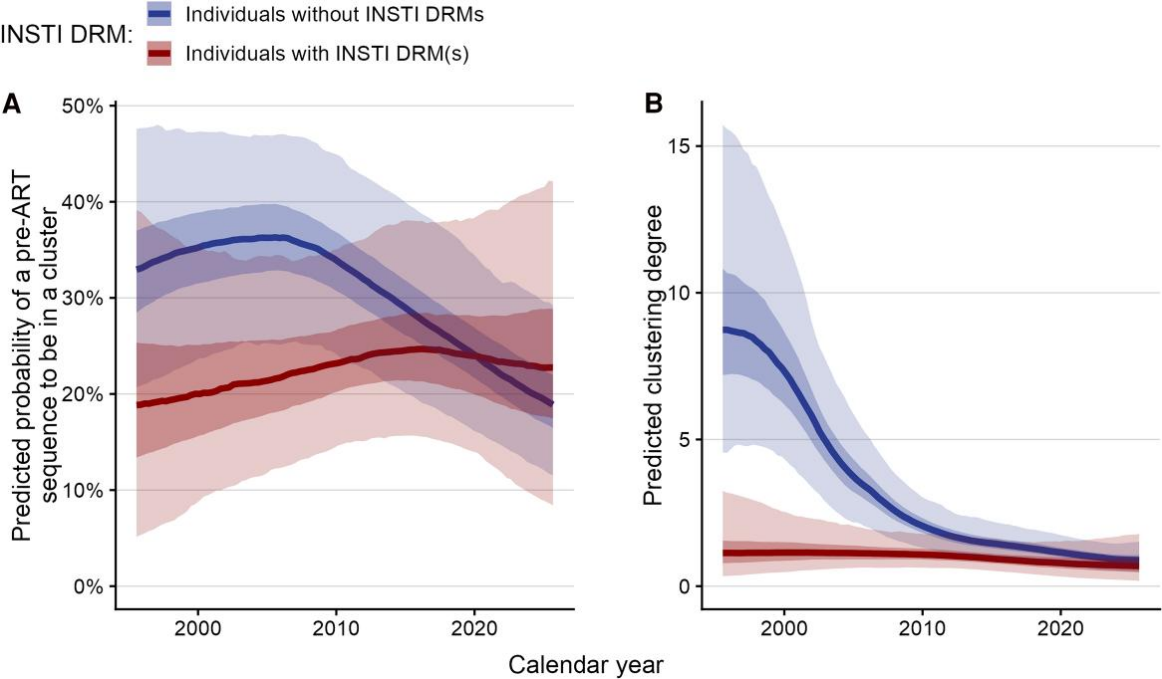
Clustering of individuals with other Swiss HIV Cohort Study (SHCS) sequences. *A*, Predicted clustering based on a bayesian logistic regression model for clustering based on protease and reverse transcriptase (PR-RT) sequence similarity with existing sequences from any SHCS participant at the time of diagnosis. Abbreviation: ART, antiretroviral therapy. *B*, Predicted clustering degree (no. of individuals to whom a newly diagnosed individual connects) at the time of diagnosis based on a negative binomial zero-inflated bayesian regression model. In both models, calendar time was modeled with a penalized spline allowed to vary by presence or absence of any integrase strand transfer inhibitor (INSTI) drug resistance mutations (DRMs) through an interaction term, while including the main effects of having any INSTI DRMs. The models were further adjusted for age and sex, including random effects for the sequencing laboratory and the SHCS center of first registration. Solid line represent the posterior median of the expected probability; darker band, the 50% credible interval; and lighter band, the 95% credible interval. Group-level effects were marginalized; uncertainty reflects the mean expectation rather than individual-level variability.

Individuals with pre-ART INSTI DRMs are less likely to connect to epidemiological clusters of SHCS participants than those without pre-ART INSTI DRMs, but they follow roughly the same trend over the past decades, with the probability of clustering being relatively stable until 2010 and decreasing since (Figure 5*A*). However, the number of SHCS participants to whom a newly diagnosed individual connects is markedly higher for those without pre-ART INSTI DRMs in the early years, but the difference becomes less pronounced over time (Figure 5*B*). When considering participants with HIV subtype B only, we observe the same dynamics (Supplementary Figure 7).

Among the individuals with pre-ART major INSTI DRMs, 4 of 11 (36.4%) have a PR-RT sequence similarity of <1% with another SHCS participant (ie, form a cluster) at the time of diagnosis (Supplementary Table 2). These clusters are small, with 3 being pairs and 1 comprising 4 individuals. None of the cluster members had viremia during an INSTI-based regimen before the sampling date for the sequence in this study, and there is no evidence of INSTI DRMs for those cluster members with available sequencing information (Supplementary Figures 8–11).

## DISCUSSION

In the SHCS, the probability of detecting pre-ART INSTI DRMs was low, particularly for major INSTI DRMs, which we found in only 11 of 3704 individuals with pre-ART integrase sequences. Among those, the efficacy of the currently used second-generation INSTIs dolutegravir or bictegravir would be notably reduced only in 1 individual with R263K that occurred mixed with wild-type HIV. In a subsequent sample collected 2 weeks later, R263K was not detected in the consensus sequence anymore, possibly due to purifying selection after recent HIV infection, which would be in line with findings of a UK study in which INSTI DRMs in mixed forms were observed more frequently among recently infected individuals than at later stages [9].

The accessory INSTI DRMs E157Q, T97A, and L74M, which are considered polymorphic [13], were most frequently detected. While their prevalence was similar to that described in an earlier systematic review [10], the increase over time reported in that review could not be seen in SHCS participants. In our analyses, we found that the probability of INSTI DRMs was higher among individuals of non-White race/ethnicity with non-B HIV subtype but did not change over time. However, as they comprise an increasing proportion among individuals with newly diagnosed HIV in Switzerland [6], increases in polymorphic pre-ART INSTI DRMs may also be observed in the SHCS in coming years.

Mbisa and colleagues [9] showed that INSTI DRMs are more common among individuals with a recent HIV infection, particularly as low-frequency variants. Given the high fitness cost, particularly of major INSTI DRMs [28, 29], they may revert or be lost due to purifying selection following HIV transmission, which represents an extremely strong genetic bottleneck. Polymorphic INSTI DRMs, which generally have low fitness cost, have been suggested as mutations emerging during viremia by selection pressure through INSTIs that persist, in contrast to major INSTI DRMs [10]. The only major INSTI DRM we observed multiple times is E138K, which has one of the lowest fitness costs among major INSTI DRMs [28]. This is in line with findings previously described by Yang et al in the SHCS [30, 31], where persistence of transmitted DRMs was associated with their impact on viral fitness.

In the analysis on epidemiological clustering, we observe that while the probability of connecting to a cluster at HIV diagnosis is similar for those with and those without pre-ART INSTI DRMs, the cluster connectivity among the former is constantly low (ie, only forming small clusters over the past 3 decades), suggesting a difference from the “typical” Swiss HIV epidemiology in the early years. After 2010, the clustering dynamics become more similar (ie, independent of pre-ART INSTI DRMs). Considering the robustness of this finding when limiting the population to those with HIV subtype B, this effect is unlikely to be confounded by origin of the SHCS participant.

The origin of the pre-ART INSTI DRMs observed in SHCS participants is unclear. Among those with pre-ART major INSTI DRMs, 4 cluster with other SHCS participants. There is neither evidence of evolutionary pressure for the selection of INSTI resistance among cluster members predating the sample where pre-ART INSTI DRMs was detected, nor evidence for the transmission of INSTI DRMs. Of note, the clusters represent only individuals within the SHCS where sequencing information is available and do not necessarily reflect the complete transmission network; clustering thus does not necessarily imply HIV transmission between individuals in a cluster [17]. In addition, 9 of the 11 individuals with pre-ART major INSTI DRMs predate the use of INSTI for ART, suggesting that in Switzerland selection pressure by INSTIs may play only a minor role by 2025. Finally, in the SHCS, the proportion of individuals on INSTI-based ART with viremia >1000 copies/mL is very low, reaching the maximum number of 34 of 7122 individuals (0.5%) at the end of 2019 (Supplementary Figure 1). Among them, even fewer have documented INSTI resistance, indicating a negligible population-level risk of HIV transmission including an HIV strain carrying INSTI DRMs among SHCS participants. This finding is consistent with previous findings by Scherrer et al [5], who showed that in 2014 in the SHCS—in contrast to older antiretroviral drug classes—the estimated population viral load among individuals with INSTI exposure was very low [5].

Our study has both limitations and strengths. While the SHCS is representative of individuals on ART in Switzerland, it may not be for those newly diagnosed, and not all people living with HIV in Switzerland participate in the SHCS. In addition, participants with pre-ART sequences were not fully representative of the entire cohort history, as sequencing was not fully implemented at cohort inception in 1988, resulting in underrepresentation of individuals entering the cohort in the very early years, particularly those with HIV transmission via injecting drug use. However, we consider it unlikely that this affected our findings, given the roughly 2 decades of pre-INSTI treatment and stable pretreatment INSTI resistance trends. There are also substantial knowledge gaps regarding the transmissibility of INSTI DRMs and their impact on viral fitness, which poses an additional challenge in interpreting pre-ART INSTI DRMs. As integrase sequences were obtained before ART initiation but not necessarily during acute infection, we cannot reliably identify transmitted resistance from among pre-ART INSTI resistance at the individual level; however, given the low prevalence of INSTI resistance and the stable population-level trends, this distinction is unlikely to affect our findings. Moreover, as INSTI DRMs may come with substantial reductions in viral fitness, transmitted INSTI resistance may even be underestimated if mutations revert before sampling. While our study relied on HIV consensus sequences generated from plasma RNA, in which APOBEC-mediated mutations are unlikely to be detected at frequencies high enough for mutation calling [15], we cannot formally exclude the possibility that individual DRMs originated from APOBEC editing; however, this is unlikely to affect our findings, given the already low prevalence of INSTI DRMs and given that APOBEC-mediated mutations would be expected to occur in the context of broader G-to-A hypermutation, including premature stop codons, which would render a viral sequence inviable [14]. However, the current study is among the largest by number of available pre-ART integrase sequences, as well as by the time covered, allowing for assessment of trends in mutation prevalence from well before to more than a decade of INSTI-based ART. Combined with the rich and densely sampled data available in the SHCS, including sequencing information, this allows for a comprehensive study on pre-ART INSTI resistance.

The implications of our findings for other settings should be interpreted cautiously. The SHCS represents a well-resourced setting with dense longitudinal follow-up, frequent viral load monitoring, broad access to individualized ART, a biobank, and a linked resistance database. In resource-limited settings with a programmatic approach to ART, higher HIV prevalence, less frequent viral load monitoring, limited access to resistance testing, and fewer treatment options, the epidemiological conditions for emergence and onward transmission of INSTI DRMs may differ substantially. In particular, prolonged viremia during INSTI-based ART may allow accumulation of INSTI DRMs and selection of viral strains with compensatory amino acid substitutions, improving viral fitness and thereby increasing the probability of emerging pretreatment INSTI resistance at the population level [4]. However, while our findings may thus not be directly transferable to resource-limited settings, integrating resistance tests from observational cohorts into HIV drug resistance surveillance strategies may help overcome some limitations that purely cross-sectional surveillance methods face, such as providing longitudinal denominators, clinical covariates, and the possibility of follow-up after the detection of drug resistance. Approaches combining sentinel surveillance studies with longitudinal cohorts or programmatic databases would align with the recent World Health Organization integrated drug resistance action framework [32].

In conclusion, in the SHCS, where INSTI-based regimens have been used for more than a decade by thousands of individuals, the proportion of those with INSTI DRMs before starting ART has remained constant at low levels and has not increased compared with the pre-INSTI era, demonstrating that pretreatment INSTI resistance can be successfully prevented. However, drug resistance surveillance is essential. as the risk of emergent INSTI resistance is currently increasing globally [2, 4, 33], which may result in more HIV transmission including HIV strains carrying INSTI DRMs. Real-time surveillance in observational HIV cohorts with a linked drug resistance database offers a valuable tool for a representative assessment of pre-ART drug resistance, which will also help ensure the sustainability of INSTI-based ART in the coming years.

## Supplementary Material

ofag435_Supplementary_Data

## Supplementary Data

Supplementary materials are available at *Open Forum Infectious Diseases* online. Consisting of data provided by the authors to benefit the reader, the posted materials are not copyedited and are the sole responsibility of the authors, so questions or comments should be addressed to the corresponding author.

## References

[1] Loosli T, Hossmann S, Ingle SM, et al HIV-1 drug resistance in people on dolutegravir-based antiretroviral therapy: a collaborative cohort analysis. Lancet HIV 2023; 10:e733–41. 37832567 10.1016/S2352-3018(23)00228-X PMC10913014

[2] World Health Organization (WHO) . HIV Drug resistance: brief report 2024. Geneva, Switzerland: World Health Organization; 2024.

[3] Loosli T, Moore CB, Buzaalirwa L, et al Drug resistance in people with viremia on dolutegravir-based antiretroviral therapy in sub-Saharan Africa: the DTG RESIST study. Clin Infect Dis 2025; 81: e128–31. 40391923 10.1093/cid/ciaf204 PMC12596406

[4] Loosli T, Han N, Hauser A, et al Predicted dolutegravir resistance in people living with HIV in South Africa during 2020–35: a modelling study. Lancet Glob Health 2025; 13:e698–706. 40155107 10.1016/S2214-109X(24)00553-9

[5] Scherrer AU, Yang WL, Kouyos RD, et al Successful prevention of transmission of integrase resistance in the Swiss HIV cohort study. J Infect Dis 2016; 214:399–402.. 27130429 10.1093/infdis/jiw165

[6] Duran Ramirez JJ, Ballouz T, Nguyen H, et al Increasing frequency and transmission of HIV-1 non-B subtypes among men who have sex with men in the Swiss HIV cohort study. J Infect Dis 2022; 225:306–16. 34260728 10.1093/infdis/jiab360

[7] Mielczak K, Serwin K, Urbańska A, et al Frequency of major transmitted integrase resistance in Poland remains low despite change in subtype variability. Viruses 2024; 16:1597. 39459930 10.3390/v16101597 PMC11512334

[8] de Salazar A, Viñuela L, Fuentes A, et al Transmitted drug resistance to integrase-based first-line human immunodeficiency virus antiretroviral regimens in Mediterranean Europe. Clin Infect Dis 2023; 76:1628–35. 36571282 10.1093/cid/ciac972

[9] Mbisa JL, Ledesma J, Kirwan P, et al Surveillance of HIV-1 transmitted integrase strand transfer inhibitor resistance in the UK. J Antimicrob Chemother 2020; 75:3311–8. 32728703 10.1093/jac/dkaa309 PMC7566560

[10] Bailey AJ, Rhee SY, Shafer RW. Integrase strand transfer inhibitor resistance in integrase strand transfer inhibitor-naive persons. AIDS Res Hum Retroviruses 2021; 37:736–43. 33683148 10.1089/aid.2020.0261 PMC8665799

[11] Scherrer AU, Traytel A, Braun DL, et al Cohort profile update: the Swiss HIV Cohort Study (SHCS). Int J Epidemiol 2022; 51:33–4J. 34363666 10.1093/ije/dyab141

[12] Zeeb M, Frischknecht P, Balakrishna S, et al Addressing data management and analysis challenges in viral genomics: the Swiss HIV Cohort Study viral next generation sequencing database. PLOS Digit Health 2025; 4:e0000825. 40257980 10.1371/journal.pdig.0000825 PMC12011223

[13] Liu TF, Shafer RW. Web resources for HIV type 1 genotypic-resistance test interpretation. Clin Infect Dis 2006; 42:1608–18. 16652319 10.1086/503914 PMC2547473

[14] Li Y, Etemad B, Dele-Oni R, et al Drug resistance mutations in HIV provirus are associated with defective proviral genomes with hypermutation. AIDS 2021; 35:1015–20. 33635848 10.1097/QAD.0000000000002850 PMC8102365

[15] Tzou PL, Pond K, Avila-Rios SL, et al Analysis of unusual and signature APOBEC-mutations in HIV-1 *pol* next-generation sequences. PLoS One 2020; 15:e0225352. 32102090 10.1371/journal.pone.0225352 PMC7043932

[16] Bürkner PC . Bayesian item response modeling in R with brms and stan. J Stat Softw 2021; 100:1 54.

[17] Labarile M, Loosli T, Zeeb M, et al Quantifying and predicting ongoing human immunodeficiency virus type 1 transmission dynamics in Switzerland using a distance-based clustering approach. J Infect Dis 2023; 227:554–64. 36433831 10.1093/infdis/jiac457

[18] Li M, Passos DO, Shan Z, et al Mechanisms of HIV-1 integrase resistance to dolutegravir and potent inhibition of drug-resistant variants. Sci Adv 2023; 9:eadg5953. 37478179 10.1126/sciadv.adg5953 PMC11803526

[19] Stanford University . HIV drug resistance database—INSTI resistance notes. 2025. Available at: https://hivdb.stanford.edu/dr-summary/resistance-notes/INSTI/. Accessed 14 October 2025.

[20] Rhee SY, Taylor J, Fessel WJ, et al HIV-1 protease mutations and protease inhibitor cross-resistance. Antimicrob Agents Chemother 2010; 54:4253–61. 20660676 10.1128/AAC.00574-10 PMC2944562

[21] Rhee SY, Varghese V, Holmes SP, et al Mutational correlates of virological failure in individuals receiving a WHO-recommended tenofovir-containing first-line regimen: an international collaboration. EBioMedicine 2017; 18:225–35. 28365230 10.1016/j.ebiom.2017.03.024 PMC5405160

[22] Svarovskaia ES, Feng JY, Margot NA, et al The A62V and S68G mutations in HIV-1 reverse transcriptase partially restore the replication defect associated with the K65R mutation. J Acquir Immune Defic Syndr 2008; 48:428–36. 18614922 10.1097/QAI.0b013e31817bbe93

[23] Porter DP, Toma J, Tan Y, et al Clinical outcomes of virologically-suppressed patients with pre-existing HIV-1 drug resistance mutations switching to rilpivirine/emtricitabine/tenofovir disoproxil fumarate in the SPIRIT study. HIV Clin Trials 2016; 17:29–37. 26899540 10.1080/15284336.2015.1115585

[24] Azijn H, Tirry I, Vingerhoets J, et al TMC278, a next-generation nonnucleoside reverse transcriptase inhibitor (NNRTI), active against wild-type and NNRTI-resistant HIV-1. Antimicrob Agents Chemother 2010; 54:718–27. 19933797 10.1128/AAC.00986-09 PMC2812151

[25] Rimsky L, Vingerhoets J, van Eygen V, et al Genotypic and phenotypic characterization of HIV-1 isolates obtained from patients on rilpivirine therapy experiencing virologic failure in the phase 3 ECHO and THRIVE studies: 48-week analysis. J Acquir Immune Defic Syndr 2012; 59:39–46. 22067667 10.1097/QAI.0b013e31823df4da

[26] Tambuyzer L, Vingerhoets J, Azijn H, et al Characterization of genotypic and phenotypic changes in HIV-1-infected patients with virologic failure on an etravirine-containing regimen in the DUET-1 and DUET-2 clinical studies. AIDS Res Hum Retroviruses 2010; 26:1197–205. 20854144 10.1089/aid.2009.0302

[27] Stanford University HIV Drug Resistance Database . NNRTI resistance notes. Available at: https://hivdb.stanford.edu/dr-summary/resistance-notes/NNRTI/. Accessed 28 October 2025.

[28] Shafer R . Phenotypic impact of INSTI-associated DRMs. Presented at: XXXI International Workshop on HIV Drug Resistance and Treatment Strategies. 2025. Available at: https://www.hivresistance.co.za/wp-content/uploads/2025/09/Tues_Phenotypic-Impact-of-INSTI-Associated-DRMs_Shafer.pdf. Accessed 30 October 2025.

[29] Tao K, Rhee SY, Chu C, et al Treatment emergent dolutegravir resistance mutations in individuals naïve to HIV-1 integrase inhibitors: a rapid scoping review. Viruses 2023; 15:1932. 37766338 10.3390/v15091932 PMC10536831

[30] Yang WL, Kouyos RD, Böni J, et al Persistence of transmitted HIV-1 drug resistance mutations associated with fitness costs and viral genetic backgrounds. PLoS Pathog 2015; 11:1–13. 10.1371/journal.ppat.1004722 PMC4370492 25798934

[31] Kühnert D, Kouyos R, Shirreff G, et al Quantifying the fitness cost of HIV-1 drug resistance mutations through phylodynamics. PLoS Pathog 2018; 14:e1006895. 29462208 10.1371/journal.ppat.1006895 PMC5877888

[32] World Health Organisation (WHO) . Integrated drug resistance action framework for HIV, hepatitis B and C and sexually transmitted infections, 2026–2030. Geneva, Switzerland: World Health Organization, 2025.

[33] McClure M, Gandhi M. Threat of HIV and tuberculosis drug resistance after US funding cuts. Lancet Infect Dis 2025; 25:e256–7. 40122094 10.1016/S1473-3099(25)00209-9

