# Supplementary material for: Pretreatment Resistance to Integrase Inhibitors in the Swiss HIV Cohort Study From 1995 to 2025
Source: Open Forum Infect Dis. 2026 Jul 17;13(8):ofag435. doi: 10.1093/ofid/ofag435 (PMC13456085; doi:10.1093/ofid/ofag435)
Supplement: Supplementary file 1 [file ofag435_supplementary_data.docx]

# Supplement

## Statistical models and prior specification

The following Bayesian logistic regression models were fitted:

i) Outcome: any INSTI DRM(s); covariables: calendar time (scaled, spline), age (scaled), sex; random effects: laboratory, SHCS center of registration.
ii) Outcome any INSTI DRM(s) at non-polymorphic positions; covariables: calendar time (scaled, spline), age (scaled), sex; random effects: laboratory, SHCS center of registration.
iii) Outcome any major INSTI DRM(s); covariables: calendar time (scaled, spline), age (scaled), sex; random effects: laboratory, SHCS center of registration.
iv) Outcome any polymorphic INSTI DRM(s); covariables: calendar time (scaled, spline by HIV subtype and ethnicity), HIV subtype and ethnicity, age (scaled), sex; random effects: laboratory, SHCS center of registration.
v) Outcome any INSTI DRM(s); covariables: calendar time (scaled, spline by HIV subtype and ethnicity), HIV subtype and ethnicity, age (scaled), sex; random effects: laboratory, SHCS center of registration.
vi) Outcome any INSTI DRM(s); covariables: calendar time (scaled, linear, interaction with HIV subtype and ethnicity), HIV subtype and ethnicity, age (scaled), sex; random effects: laboratory, SHCS center of registration.
vii) Outcome any INSTI DRM(s) at non-polymorphic positions including HIV subtype and ethnicity covariables: calendar time (scaled, linear, interaction with HIV subtype and ethnicity), HIV subtype and ethnicity, age (scaled), sex; random effects: laboratory, SHCS center of registration.

viii-xii)	Outcome presence or absence of specific INSTI DRMs observed at least 10 times (E157Q, G163R/K, L74M, S153A/Y/F, T97A); covariables: scaled calendar time (spline), age (scaled), sex; random effects: laboratory, SHCS center of registration.

xiii) Outcome any clustering with other SHCS sequences; covariables: calendar time (scaled, spline by presence or absence of any INSTI DRM(s)), presence or absence of any INSTI DRM(s), age (scaled), sex; random effects: laboratory, SHCS center of registration.
xiv) Outcome any clustering with other SHCS sequences (restricted to participants with HIV subtype B); covariables: calendar time (scaled, spline by presence or absence of any INSTI DRM(s)), presence or absence of any INSTI DRM(s), age (scaled), sex; random effects: laboratory, SHCS center of registration.

The following Bayesian zero inflated negative binomial models were fitted:

xv) Outcome node degree; covariables: calendar time (scaled, spline by presence or absence of any INSTI DRM(s)), presence or absence of any INSTI DRM(s), age (scaled), sex; random effects: laboratory, SHCS center of registration.
xvi) Outcome node degree (restricted to participants with HIV subtype B); covariables: calendar time (scaled, spline by presence or absence of any INSTI DRM(s)), presence or absence of any INSTI DRM(s), age (scaled), sex; random effects: laboratory, SHCS center of registration

Priors were specified to be weakly informative for all model parameters. Priors on intercept were normal(-4, 1.5) for models i-xii, normal(0, 1.5) for models xiii and xiv, and normal(1, 2) for models xv and xvi. For all models, regression coefficients for the fixed effects age and sex (and subtype and ethnicity for models v-vii; and presence or absence of INSTI DRM for models xiii-xvi) had normal(0, 1.5) priors. Random-effect standard deviations for sequencing laboratory and SHCS center of first registration followed half-Student-t distributions with 3 degrees of freedom and a scale of 1. Spline smoothness parameters (sds) were given the same half-Student-t(3, 0, 1) prior. Additional parameters in the zero-inflated negative binomial models (xv and xvi) were overdispersion (shape) parameter which had a lognormal(0, 0.5) prior, and the zero-inflation probability which was assigned a normal(0.16, 1) prior.

## Supplemental figures and tables

Supplemental figure 1: **SHCS participants with viremia on any INSTI-based ART.** Blue area corresponds to the number of individuals on INSTI-based ART (i.e., regimen containing raltegravir, elvitegravir, dolutegravir, bictegravir, or cabotegravir), updated every 90 days. Red corresponds to SHCS participants with closest viral load (within 365 days) over 1000 copies/ml among all SHCS participants on INSTI-based ART as proportion left, and in absolute numbers on the right. Purple corresponds to those with documented INSTI resistance mutations at any timepoint before date of viral load above 1000 copies/ml + 30 days. The first datapoint corresponds to the first observation of an individual on INSTI-based ART with a virus load above 1000 copies/ml in the SHCS.

Supplemental figure 2: **Prior predictive checks**. Shown are draws from the prior predictive distribution (n=100) for each model. For Bayesian logistic models (Bernoulli family), blue bars (y) correspond to observed counts in the dataset, black dot and line (yrep) correspond to median and 95% interval from prior predictive distributions. For zero-inflated negative binomial models, outcome (count) densities are shown on log log scale; observed counts (y) in dark red, and draws from prior predictive distributions (yrep) in salmon.

Supplemental table 1 **SHCS population by availability of pre-ART sequences**. Data availability is lower in the early years of the SHCS.

|  | **Total SHCS population** | | **Registration after 1995** | |
| --- | --- | --- | --- | --- |
|  | **No Pre-ART sequence**  (N=12157) | **With Pre-ART sequence**  (N=10194) | **No Pre-ART sequence**  (N=5868) | **With Pre-ART sequence**  (N=9297) |
| **SHCS registration date** | 1994.5 [1990.3 - 2005.3] | 2007.0 [1999.3 - 2013.2] | 2006.0 [1999.1 - 2016.0] | 2008.0 [2001.3 - 2013.7] |
| **Sex** |  |  |  |  |
| Female | 3374 (27.8%) | 2645 (25.9%) | 1768 (30.1%) | 2346 (25.2%) |
| Male | 8783 (72.2%) | 7549 (74.1%) | 4100 (69.9%) | 6951 (74.8%) |
| **Age at SHCS registration** | 34.0 [29.0 - 42.0] | 36.0 [30.0 - 45.0] | 38.0 [32.0 - 46.0] | 37.0 [31.0 - 46.0] |
| **Education** |  |  |  |  |
| Apprenticeship | 3280 (27.0%) | 4352 (42.7%) | 1992 (33.9%) | 3989 (42.9%) |
| Mandatory | 1633 (13.4%) | 1745 (17.1%) | 1085 (18.5%) | 1564 (16.8%) |
| University | 1155 (9.5%) | 1171 (11.5%) | 999 (17.0%) | 1150 (12.4%) |
| Higher professional education | 684 (5.6%) | 801 (7.9%) | 512 (8.7%) | 776 (8.3%) |
| None | 674 (5.5%) | 694 (6.8%) | 488 (8.3%) | 651 (7.0%) |
| Higher technical/commercial school | 426 (3.5%) | 656 (6.4%) | 315 (5.4%) | 626 (6.7%) |
| Bachelor | 464 (3.8%) | 479 (4.7%) | 369 (6.3%) | 449 (4.8%) |
| Other | 51 (0.4%) | 25 (0.2%) | 27 (0.5%) | 23 (0.2%) |
| Missing | 3790 (31.2%) | 271 (2.7%) | 81 (1.4%) | 69 (0.7%) |
| **Ethnicity** |  |  |  |  |
| White | 7269 (59.8%) | 7870 (77.2%) | 4118 (70.2%) | 7101 (76.4%) |
| Black | 1125 (9.3%) | 1382 (13.6%) | 1083 (18.5%) | 1368 (14.7%) |
| Hispano-American | 330 (2.7%) | 410 (4.0%) | 318 (5.4%) | 406 (4.4%) |
| Asian | 275 (2.3%) | 390 (3.8%) | 248 (4.2%) | 385 (4.1%) |
| Other | 37 (0.3%) | 29 (0.3%) | 28 (0.5%) | 22 (0.2%) |
| Unknown/Missing | 3121 (25.7%) | 113 (1.1%) | 73 (1.2%) | 15 (0.2%) |
| **Region of origin** |  |  |  |  |
| Central Europe | 8514 (70.0%) | 6798 (66.7%) | 3303 (56.3%) | 6013 (64.7%) |
| Mediterian Europe | 1271 (10.5%) | 956 (9.4%) | 656 (11.2%) | 888 (9.6%) |
| Central Africa | 388 (3.2%) | 412 (4.0%) | 313 (5.3%) | 410 (4.4%) |
| Eastern Africa | 394 (3.2%) | 399 (3.9%) | 331 (5.6%) | 395 (4.2%) |
| South America | 381 (3.1%) | 371 (3.6%) | 315 (5.4%) | 361 (3.9%) |
| Western Africa | 259 (2.1%) | 321 (3.1%) | 233 (4.0%) | 318 (3.4%) |
| South Asia & Polynesia | 189 (1.6%) | 267 (2.6%) | 157 (2.7%) | 264 (2.8%) |
| East Eurpope & Russia | 175 (1.4%) | 165 (1.6%) | 149 (2.5%) | 164 (1.8%) |
| Other* | 586 (4.8%) | 505 (5.0%) | 411 (7.0%) | 484 (5.2%) |
| **Mode of HIV acquisition** |  |  |  |  |
| homosexual contacts | 4504 (37.0%) | 4509 (44.2%) | 2385 (40.6%) | 4255 (45.8%) |
| heterosexual contacts | 3538 (29.1%) | 3794 (37.2%) | 2188 (37.3%) | 3583 (38.5%) |
| i.v.drug use (with needle sharing) | 2456 (20.2%) | 892 (8.8%) | 644 (11.0%) | 621 (6.7%) |
| i.v. drug use or sexual contacts | 1055 (8.7%) | 469 (4.6%) | 247 (4.2%) | 322 (3.5%) |
| unknown/inconclusive | 284 (2.3%) | 339 (3.3%) | 199 (3.4%) | 331 (3.6%) |
| Other** | 320 (2.6%) | 191 (1.9%) | 205 (3.5%) | 185 (2.0%) |
| **Center of registration** |  |  |  |  |
| Zurich | 4060 (33.4%) | 4063 (39.9%) | 1744 (29.7%) | 3664 (39.4%) |
| Lausanne | 2170 (17.8%) | 1468 (14.4%) | 1134 (19.3%) | 1373 (14.8%) |
| Geneva | 2184 (18.0%) | 1036 (10.2%) | 915 (15.6%) | 955 (10.3%) |
| Bern | 1425 (11.7%) | 1426 (14.0%) | 743 (12.7%) | 1302 (14.0%) |
| Basel | 1261 (10.4%) | 1182 (11.6%) | 769 (13.1%) | 1080 (11.6%) |
| StGallen | 648 (5.3%) | 722 (7.1%) | 302 (5.1%) | 635 (6.8%) |
| Lugano | 409 (3.4%) | 297 (2.9%) | 261 (4.4%) | 288 (3.1%) |

Groups with less than 1% of the total population have been grouped together as ‘other’.

*: North America (n=176, 0.8%), Middle East (n=167, 0.7%), UK and Baltic (n=165, 0.7%), Carribean (n=154, 0.7%), Maghreb (n=111, 0.5%), Southern Africa (n=82, 0.4%), India (n=73, 0.3%), Central America (n=63, 0.3%), Asia (n=53, 0.2%), Unknown (n=24, 0.1%), Oceania (n=18, 0.1%), and Missing (n=5, 0%)

**: other blood products (e.g.transfusions) (n=159, 0.7%), perinatal transmission (n=149, 0.7%), other sources (n=146, 0.7%), clotting factors against hemophilia (n=53, 0.2%), and Missing (n=4, 0%)

Supplemental figure 3: **Availability of HIV sequences in the Swiss HIV Resistance Database**. A) Cumulative number of HIV sequences by ART status of the sampled individual in the resistance database linked to the SHCS. B) Conditional density estimate of integrase availability among pre-ART RNA sequences by sequence sampling year. C) Number of individuals per year with a pre-ART integrase sequence included in this study. In case of multiple sequences per individual, the date of the first available sequence was considered.

Supplemental figure 4: **Population** **trend in INSTI DRMs observed ≥ 10 times**. Bayesian regression models were fitted separately for each INSTI DRM observed more than 10 times. Calendar time was modelled with a penalized spline and the model adjusted for age and sex, including random effects for sequencing laboratory and SHCS center of first registration. The solid line shows the posterior median of the expected probability; the darker band is the 50% credible interval and the lighter band the 95% credible interval. Group-level effects were marginalized; uncertainty reflects the mean expectation rather than individual-level variability.

Supplemental table 2: **Resistance mutations for all drug classes in participants with major INSTI DRM(s).** Additional pre-ART sequences from later timepoints were available for three participants.

| **Participant** | **Region of origin** | **HIV subtype** | **Sample date** | **PI Major** | **PI Accessory** | **NRTI** | **NNRTI** | **INSTI Major** | **INSTI Accessory** | **BIC/DTG resistance** | **CAB resistance** |
| --- | --- | --- | --- | --- | --- | --- | --- | --- | --- | --- | --- |
| **1** | Central Europe | A | 17.08.2000 | None | L33F | None | None | N155S | S153Y | Low-Level | Low-Level |
| **2** | Central Europe | B | 13.12.2001 | None | None | None | E138K | E138K | None | Potential Low-Level | Low-Level |
| **3^#^** | Central Europe | B | 26.06.2003 | None | None | K219R | P236L | E92G, G140A | None | Potential Low-Level | Low-Level |
| **4** | Central Europe | B | 26.09.2005 | None | None | S68G, T215D | None | E138K | None | Potential Low-Level | Low-Level |
| **5*** | Mediterranean Europe | C | 15.03.2006 | None | None | None | None | F121FY | None | Potential Low-Level | Low-Level |
| **6^#^** | South America | B | 08.08.2007 | None | None | T215D | None | Q148R | None | Low-Level | High-Level |
| **7** | Central Europe | B | 08.08.2007 | None | None | None | None | E138K | None | Potential Low-Level | Low-Level |
| **8^#^** | Central Europe | Rec. | 04.01.2010 | None | None | None | None | T66I | None | Susceptible | Potential Low-Level |
| **9**** | Central Africa | D | 23.02.2010 | None | None | None | None | E138K | None | Potential Low-Level | Low-Level |
| **10***, #** | Central Europe | B | 21.03.2014 | None | None | None | None | R263RK | None | Intermediate | High-Level |
| **11** | Maghreb | B | 17.12.2018 | D30N | K43T, N88D | None | E138EA | E138K | None | Potential Low-Level | Low-Level |

*	subsequent sample from 10.11.2006, 240 days after study sample: no INSTI DRMs detected

**	subsequent sample from 26.08.2010, 184 days after study sample: E138K detected

***	subsequent sample from 02.04.2014, 12 days after study sample: no INSTI DRMs detected

\#	clusters with one or more SHCS participants at timepoint of diagnosis

Supplemental figure 5: **Trends in observing any polymorphic INSTI DRM(s) over time**. Polymorphic INSTI DRM(s) included E157Q (N=73), T97A (N=45), and L74M (N=36). A Bayesian regression model was fitted for individuals having any polymorphic INSTI DRM. Calendar time was modelled with a penalized spline and the model adjusted for age and sex, including random effects for sequencing laboratory and SHCS center of first registration. The solid line shows the posterior median of the expected probability; the darker band is the 50% credible interval and the lighter band the 95% credible interval. Group-level effects were marginalized; uncertainty reflects the mean expectation rather than individual-level variability.

Supplemental table 3: **Model estimates for interaction between calendar time and HIV subtype and ethnicity**. We modelled the probability of observing any INSTI DRM(s) using a Bayesian logistic regression model, including calendar time as a linear covariable with an interaction term with HIV-subtype and ethnicity. The model was further adjusted for age and sex, and included random effects for the center where the individual was first registered and the lab where the sequence was generated. Log odds were exponentiated and rounded to 3 digits.

| **Parameter** |  | **Estimate**  **(Odds ratio)** | **Lower confidence interval (95%)** | **Upper confidence interval (95%)** |
| --- | --- | --- | --- | --- |
| **Intercept** |  | 0.04 | 0.019 | 0.075 |
| **Age (scaled)** |  | 1.069 | 0.929 | 1.228 |
| **Sex (ref: Male)** | Female | 1.219 | 0.851 | 1.733 |
| **Calendar time (scaled)** |  | 0.856 | 0.624 | 1.18 |
| **Subtype & Ethnicity (ref: B, White)** | No B, White | 1.663 | 1.166 | 2.396 |
|  | No B, Other | 3.203 | 2.116 | 4.829 |
|  | B, Other | 1.616 | 0.854 | 2.898 |
| **Interaction terms** | Calendar time : no B, White | 0.863 | 0.606 | 1.22 |
|  | Calendar time : no B, Other | 0.935 | 0.636 | 1.372 |
|  | Calendar time : B, Other | 1.008 | 0.515 | 2.056 |

Supplemental figure 6: **Predicted time trends of the non-polymorphic subset among INSTI DRMs.** Bayesian logistic regression model for presence or absence of any non-polymorphic INSTI DRM(s), including calendar time as a spline by subtype and ethnicity. The model was further adjusted for age and sex, and included random effects for the center where the individual was first registered and the lab where the sequence was generated. Predictions are based on 1000 random draws from the expected posterior distribution; the solid line corresponds to the median estimate, inner shaded area to the interquartile range, and outer shaded area to the 95% range across estimates.

Supplemental figure 7: **Clustering of individuals with HIV subtype B with other SHCS sequences**. A) Predicted clustering based on a Bayesian logistic regression model for clustering based on PR-RT sequence similarity with existing sequences from any SHCS participant at time of diagnosis. B) Predicted clustering degree (number of individuals a newly diagnosed individual connects to) at time of diagnosis based on a negative binomial zero inflated Bayesian regression model.

In both models, the population was restricted to individuals with HIV subtype B; calendar time was modelled with a penalized spline allowed to vary by presence or absence of any INSTI DRM(s) through an interaction term, while including main effects of having any INSTI DRM(s). The models were further adjusted for age and sex, including random effects for sequencing laboratory and SHCS center of first registration. The solid line shows the posterior median of the expected probability; the darker band is the 50% credible interval and the lighter band the 95% credible interval. Group-level effects were marginalized; uncertainty reflects the mean expectation rather than individual-level variability.

Supplemental figure 8: All individuals in the cluster formed at diagnosis of study participant 3 with the pre-ART major INSTI DRMs E92G and G140A. Sequencing results are only shown if integrase is covered; results from proviral sequences are marked with a star. Viral loads dates, treatment start and stops, discontinuation dates, and date of resistance testing have been randomly shifted by up to 14 days. Viral loads on log10 scale above detection limit have a random noise up to 0.2 added or subtracted, applied to each value individually. For SHCS participant 4, regimen durations less than 90 days are not labelled.

Supplemental figure 9: All individuals in the cluster formed at diagnosis of study participant 6 with the pre-ART major INSTI DRM Q148R. Sequencing results are only shown if integrase is covered; results from proviral sequences are marked with a star. Viral loads dates, treatment start and stops, discontinuation dates, and date of resistance testing have been randomly shifted by up to 14 days. Viral loads on log10 scale above detection limit have a random noise up to 0.2 added or subtracted, applied to each value individually.

Supplemental figure 10: All individuals in the cluster formed at diagnosis of study participant 8 with the pre-ART major INSTI DRM T66I. Sequencing results are only shown if integrase is covered; results from proviral sequences are marked with a star. Viral loads dates, treatment start and stops, discontinuation dates, and date of resistance testing have been randomly shifted by up to 14 days. Viral loads on log10 scale above detection limit have a random noise up to 0.2 added or subtracted, applied to each value individually.

Supplemental figure 11: All individuals in the cluster formed at diagnosis of study participant 10 with the pre-ART major INSTI DRM R263RK. Sequencing results are only shown if integrase is covered; results from proviral sequences are marked with a star. Viral loads dates, treatment start and stops, discontinuation dates, and date of resistance testing have been randomly shifted by up to 14 days. Viral loads on log10 scale above detection limit have a random noise up to 0.2 added or subtracted, applied to each value individually.
